# ‘How deep are your pockets?’ Autoethnographic reflections on the cost of raising a child with autism

**DOI:** 10.4102/ajod.v7i0.356

**Published:** 2018-03-27

**Authors:** Mary G. Clasquin-Johnson, Michel Clasquin-Johnson

**Affiliations:** 1 Department of Inclusive Education, University of South Africa, South Africa; 2 Department of Religious Studies and Arabic, University of South Africa, South Africa

## Abstract

**Background:**

In this article, we reflected on our experience of the cost of parenting a child with autism, including our ongoing search for educational and therapeutic intervention.

**Objectives:**

We aimed to give an academic insight into the state of autism education and care in South Africa as seen by us, with special attention to its cost and sustainability.

**Methods:**

Using evocative autoethnography as storied scholarship together with critical autism studies, we reflected on stories of the past 5 years since our son’s diagnosis.

**Results:**

Our experiences agree with international studies that establish autism as the most expensive disability. In addition to the high costs of diagnosis, existing intervention and support approaches are unaffordable for the majority of South Africans. We recommend that teachers should be trained to participate in early screening and diagnosis, as well as co-therapists, to strengthen the implementation of inclusive education.

**Conclusion:**

The kind of autism intervention currently offered in South Africa is financially and socially unsustainable. Instead of positioning autism as an individual or family dilemma, it should be addressed as an educational and societal issue. Future research should explore cost-effective options for a developing country context, while promoting best practice within inclusive settings.

## Introduction

In this article, we reflect on our lived experience of parenting a child with autism, specifically our search for educational and therapeutic intervention. We share stories of critical moments in our journey using qualitative inquiry and evocative autoethnography. Each could be an article in its own right, and may yet become that, but here we focus on the financial aspect. Drawing on a critical autism studies perspective (Davidson & Orsini 2013), we reflect on the cost of raising our child. We soon discovered that autism is positioned as the most expensive disability for which parents need impossibly deep pockets. This is known to be true internationally (Cidav et al. 2013; Dillenburger, McKerr & Jordan 2014; Fletcher, Markoulakis & Bryden 2012; Horlin et al. 2014; Lin 2014), but what is the case in South Africa?

### Problem statement and goals of the study

When a child manifests developmental delays, parents spend time, energy and financial resources to obtain a diagnosis (Fletcher et al. 2012; Gibson 2014; McCollum 2012). Following our son’s diagnosis of being on the autism spectrum, we were confronted with the dilemma of whether to enrol our son in a mainstream or special school. In our experience, no clear guidelines accompany this process.

Autism is regarded as the most expensive disability (Byford et al. 2016; Hall, Wright & Mills 2016). Scholars such as Dillenburger et al. (2014:135) have illustrated that the costs of rearing a child with autism in the USA is three times the cost of rearing a typically developing child. Moreover, Lokhandwala, Khanna and West-Strum (2012) have found that the lifetime cost of a single person with autism is $3.2 million. Considering that the average lifetime earning of a US citizen with a college degree is estimated at $2.8 million (Carnevale, Rose & Cheah 2011), this gives us an indication that even in a wealthy, developed country, the costs involved are staggering. How much worse can it be in a developing country? In addition, although the efficacy of early intervention in autism is uncertain (Warren et al. 2011), this is what South Africans should focus on, that is, to make it affordable and accessible to South Africans living with autism.

Autism is framed as a complex, lifelong yet invisible disability (Hoogsteen & Woodgate 2013:136; Leblanc, Richardson & Burns 2009:166) accompanied by neurological and developmental abnormalities (Lokhandwala et al. 2012:95; Wang et al. 2012:1). Consequently, parents are told that their children require costly long-term intensive remediation, therapy and biomedical intervention (Dillenburger et al. 2014). In reality, few South African families can afford this because the typical cost of school fees and therapy exceeds the average household income.

### Background statement

We are both academics with PhDs. We are not typical parents because we can assess teachers’ knowledge and skills, analyse assessment reports and ask questions. We are highly educated and privileged, and one might expect that we would be better prepared than most to deal with the experience of rearing a child with special needs. This has not proven to be the case. The cost of parenting a child with autism has proven to be overwhelming. Despite our relative privilege, we too have experienced financial strain and uncertainty.

Parents of children with autism constitute a vulnerable community (Lin 2014:243) because we will do anything to give our children the support they require, especially as we realise that they will outlive us, and need to do so independently. Studies reveal that parents of children with autism experience significant stress because of the need for closer parental supervision, the more dependent relationship with their children and more intense worry about the future (Benson et al. 2011:67, 76; Pepperell, Paynter & Gilmore 2016:5). We employ autoethnography as ‘storied scholarship’ (Boylorn & Orbe 2014:13) to capture the essence of our experience and invite the readers into our thoughts, following the example of Barua (2008).

### Literature review

Autism spectrum disorder (ASD) is the most expensive disability (Byford et al. 2016; Cimera & Burgess 2011; Hall et al. 2016). This is attributable to the cost of intensive early intervention, that is, the hourly approved medical aid rate of R440 charged for therapy and the need for small classes because of the weighting of 6 given to these learners (one learner with autism equals six ‘neurotypical’ learners). Intervention is concentrated in childhood, with the cost of care being the highest during adulthood (Cidav et al. 2013:924). Diagnosis typically happens at 3–5 years, followed by intensive therapy, which declines during the teenage years and then comes a sharp increase in adult residential and medical care (Lin 2014:247; Shimabukuro, Grosse & Rice 2008:546). Families are confronted with long-term economic stress, which is a social issue (Chasson, Harris & Neely 2007:402; Lokhandwala et al. 2012:94; Wang et al. 2012:2, 6).

## Research method and design

This qualitative inquiry combines evocative autoethnography (Boylorn & Orbe 2014; Pace 2012:7) and self-reflexivity (Anderson 2006:374, Humphreys 2005:841) to reflect our insider perspective by using ourselves as the research subjects (Ellis 2014:61). We reflect on our experiences (Morella 2008; Prince 2013:319) of the past 6 years and connect it to existing scholarship (Britton 2013:iv). We employ autoethnography as a scientific method to unpack our epistemology (Ellis 2014:50), advance social justice (Morella, 2008) and provide an insight into the cost of autism diagnosis, education and support in South Africa. We also hope that it will be of benefit to parents following a similar process (Hannekom 2012:iii), in accordance with Hemelsoet’s (2014:227) assertion that social scientists should ‘take up their responsibility by turning their scientific knowledge into a vivid leverage of discussion, action and change in the public arena’.

Our discussion below will therefore build on our personal reflection, using what Humphreys (2005:842) calls ‘vignettes’. These were separately constructed to reflect our innermost thoughts and were only brought together in a single document afterwards.

The dominant ‘medical model’ of disability pathologises disability as an individual problem for which biomedical intervention is required (Cidav et al. 2013; Leigh et al. 2016; Wang et al. 2012). It persists despite policy rhetoric following the ‘social model’, which promotes social acceptance and support (Department of Education 2001). This official new direction emphasises that it is not primarily the child who must change rather the society that must accommodate the child. Our perspective follows that of Critical Autism Studies, which calls for a strategic combination of these extremes according to the support needs of the individual (Davidson & Orsini 2013).

## Ethical considerations

Ethical clearance for this report was sought and obtained from the Ethics Committee at the College of Education at the University of South Africa (REF: 2016/08/17/1130536/09/MC). The names of the institutions, organisations or individuals are not mentioned in relation to our experiences.

## Discussion

### Story 1: Diagnosis day (D-day)

Soon after our son’s first birthday, it was evident that he was not meeting the universal milestones. Physically he progressed steadily, but his vocal development was slow. Although we struggled to accept it, we commenced speech therapy as a speech therapist was readily available at his first preschool. We hoped that he would simply catch up. We too took refuge in ‘Boys start talking a little later’ and ‘Einstein didn’t start talking until he was six’.

Given that Author 1 had been active in the field of early childhood education for 20 years and that Author 2’s second major is in Psychology, we became increasingly aware of our son’s developmental delays and eventually could no longer deny the reality of the situation. In 2011, soon after his third birthday, our son was diagnosed as being ‘on the autism spectrum’. This was the outcome of a lengthy and costly process: waiting 5 months for an initial appointment with a paediatric neurologist (at an initial cost, in 2011, of R2600), returning for a follow-up assessment 6 months later (at a cost of R700 – these rates have nearly doubled since then) and receiving a verbal diagnosis of autism but a written diagnosis that only called for ‘intensive speech therapy’ without actually mentioning the autism spectrum. Indeed, the written diagnosis was produced only after we insisted and is actually a form that allowed us to claim back some of our expenses from income tax (SARS 2016).

At the end of the second visit, the paediatric neurologist advised us to come back again after a further 6 months, with a clear suggestion that this would be the long-term pattern. Author 2 asked, ‘Why? What will she tell us? “Yes, he still has autism?” So what? All we need is the SARS form’. We have not been back. Instead, we redirected our resources and intensified our son’s therapy that had commenced a year earlier. Hannekom (2012), who undertook a South African autoethnographic study on her experience of searching for an educational niche for her son, noted that:

We always knew that he was different, but it took three and a half years of countless visits to medical professionals before he was finally diagnosed with Pervasive Developmental Delay – Not Otherwise Specified (PDD-NOS), an Autism Spectrum Disorder (ASD). Our son now had a label, but still no route map. (p. xii)

Our experience confirms Hannekom’s observation that diagnosis is not accompanied by practical advice on intervention. Although Hannekom (2012) did not focus on the cost factor, one could reasonably assume that ‘countless visits to medical professionals’ would be a costly undertaking.

The moment of diagnosis of autism has been described as a turning point in one’s life (Ellis 2014:64). So it would prove to be for us.

**Author 1**: ‘I suspected autism. My sister hinted subtly and un-subtly and still I did not want to know. I really should have known. But I wanted it to be something else, something easier. Now I feel guilty and inadequate. What do I know about autism? My training has not prepared me for this. I have only worked with neurotypical children. Yet, we are not the first family to experience this. We will handle it. We will learn whatever we need to. There’s no need to panic.’

**Author 2**: ‘I have never been diagnosed as being on the spectrum. When I was my son’s age, such a diagnosis did not even exist. Nevertheless all indications are that if it had been available, I would have been diagnosed with Asperger’s Syndrome. When my son was diagnosed, it was almost a relief. “I know this. I understand it. I can help him avoid the mistakes I made”. As an adoptive parent, I have never known the comfort of seeing a little person who resembles me physically, but now it turned out that he resembled me mentally, a realm of existence I actually treasured more. It became a standing joke in our household whenever our son’s streak of stubbornness surfaced: “Are you sure you’re not his biological father too?” I left the neurologist’s office convinced that I could handle this. I was wrong. Unlike me, our son’s symptoms included a lengthy speech delay. It would be three long years before our son started speaking beyond the level of a one-year old and another two before he would really show signs of catching up with his peers. If the diagnosis stiffened my resolve, those years of waiting, trying one approach after another, led me to consider the possibility that my son might never be the educated, self-sufficient person I want him to be one day. Not only were these approaches costly, I began worrying about long-term financial planning in the event that he would always be financially dependent on us. It remains a nagging doubt that I face every day.’

Looking back, we realise that our relatively easy acceptance of the situation was influenced by two factors: (1) our educational backgrounds and (2) the fact that this was not our first encounter with autism. Author 1 has a nephew on the autism spectrum. As previously stated, Author 2 identifies as living with Asperger’s Syndrome under the previous versions of the DSM – a classic ‘absent-minded professor’, in fact (Clasquin-Johnson 2014:6).

This contributed to the diagnosis being less overwhelming, although autism became our focal point (Hoogsteen & Woodgate 2013:136). We also recognised the benefits of a diagnosis – knowing the underlying cause of our son’s developmental delays would enable us to address them appropriately (Wang et al. 2012:2) and access resources that would remain unavailable without the imprimatur of the medical establishment (cf. Zibricky 2014).

A year later, we visited an educational psychologist. Our son was unable to complete the school readiness test, and we were given the instrument to self-administer, for which we paid her R750 over and above the regular consultation fee.

**Author 1**: ‘Over the next few days, I studied that school readiness test and actually attempted drilling him to prepare for the test the school was insisting he needed to take at the end of the year. After a single attempt to practise some of the underlying skills related to the test I abandoned it. He has always treated school and home as strictly separate and he refused to cooperate. I accepted it almost too easily since my heart was not in it anyway.’

There are indications of cynicism regarding diagnosis even among those empowered to make them. Brock (2009), for example, reports that:

Although the number of supposedly classic cases of autism may for complicated reasons be somewhat rare, in Adam’s case the psychologist made it clear that her diagnosis was primarily designed to secure a set of treatments for his particular developmental deficits. This made it especially obvious that the psychologist’s diagnosis functioned as a social rather than a strictly biomedical designation. One might be tempted to ask at this point what it would mean for Adam not to ‘really have’ autism. Like other psychological conditions, whether he ‘has it’ in some essentialist sense is much more complicated to establish than one would initially assume. (p. 11)

For this reason, ‘borderline’ cases of autism remind us in a pointed way of the social construction of disease (Dhar 2009:739).

There is an alternative route to diagnosis in South Africa, namely medical professionals in state hospitals where children younger than 6 years receive free treatment. However, there are few of them and waiting lists are extremely long (Autism South Africa 2017).

Having received the diagnosis, we scoured the Internet for information on schools and support services in our vicinity, and after finding a single option, we looked further to the entire Gauteng province.

### Story 2: Finding a school – An ongoing search

All parents face a number of complex considerations when selecting a school for their children. These considerations are even more complex when deciding upon a school for a child with autism. They include:

- the school’s geographical location and admission criteria
- curriculum, particularly whether the official curriculum is followed (and the ability of a child to progress under that curriculum)
- whether specialised teaching approaches and methodologies are employed, and if the school facilitates access to additional support and therapeutic interventions
- the school’s accreditation and registration
- the qualifications and professionalism of the teaching and managerial staff
- the class size and teacher–learner ratio
- of course, affordability, including fundraising demands.

However, because our options are limited, we consciously make compromises and even overlook serious shortcomings. Author 1 is based in a university’s Department of Inclusive Education. As a result, she is also deeply concerned about a school’s ethos – the intangible things like how sensitively and respectfully staff members treat children and parents, how welcome they feel and whether assessment feedback is delivered with empathy.

**Author 1**: ‘Shortly after our son’s diagnosis, I seriously considered giving up my career to home school him. After all, I was a qualified early childhood teacher. I soon realised that since he would still require the additional therapeutic support, we would simply not be able to afford to live on one salary.’ (cf. Baker & Drapela 2010; Ouyang et al. 2014)

**Author 2**: ‘My academic career was well on track. But would I have applied for my full professorship, and later my NRF rating, the moment I fulfilled the minimum requirements, if not for the high financial demands? Perhaps not.’

Today in South Africa, few middle class parents opt for public schooling, as quality is perceived as very poor (Maodzwa-Taruvinga & Cross 2012; Spaull 2013). Instead, both fully private schools and the uniquely South African semi-privatised construct ‘former Model-C school’ serve this population. Therefore, we often remind ourselves that even if our son was neurotypical, he would still attend a private school.

The general perception of public schooling in this country does not, however, extend to those public schools that specialise in children with disabilities. These are generally highly regarded (Autism South Africa 2017). We considered this system of education, and we were on a 3-year-long waiting list for placement at a public special school for autism.

**Author 2**: ‘We visited the school and met the educational psychologist. But her opening statement “if your son comes here, he will never matriculate”, led us to eliminate the institution as a feasible option. I can accept that my son has limitations. I cannot accept those limitations being decided for him at the age of four, when they had not even assessed him.’

This school and similar institutions around the country have since come under scrutiny for not presenting the official South African curriculum. Instead, they focus on teaching manual skills such as gardening, packing grocery shelves and washing cars. There is no shortage of these skills in the country, and in our opinion, these children are being prepared for lifelong financial dependence. This issue relates to the findings of a 3-year-long investigation on curriculum adaptation for learners with autism conducted by Author 1 and will be further explored in a follow-up article.

This thrust us firmly into the private sector where fees are high (between R5000 and R7000 per month in 2015–2016) even while accreditation is non-existent. As we soon found out, the high fees do not guarantee quality education.

Some independent special schools insist upon ‘facilitators’ being appointed and paid by parents in addition to the school fees. When confronted with this requirement, we realised that it would be for the teachers’ convenience and not our son’s.

**Author 2**: ‘We walked away and enrolled our son at a school where facilitators were not required, only to discover that some children there also had facilitators: those whose behaviour needed to be managed. This appears to be a hidden cost, as we were not informed about this upon initial enrolment. Some parents were paying up to R5000 per month, roughly doubling the school fee.’

To date we have moved schools five times, an average of once a year. The schools have included a mainstream preschool, a specialised school for autism, two special schools and a small mainstream primary school. Each school promised certain services and each has contributed to our son’s development and learning. Each has also fallen short of their promises. We believe that parents often know when a particular school has done what they can and when it is time to move on. However, how soon they act on this will vary. Moving on also requires a viable alternative, which is seldom available.

Our main considerations are whether teachers are qualified and skilled, whether the school follows the official curriculum and whether our son is able to cope with the daily demands of the school. However, it comes at a cost because teachers who possess higher levels of training and qualifications demand higher remuneration, leading to higher school fees. While these issues relate to the right to meaningful educational provision as articulated in international legal frameworks (Marshall & Goodall 2015), in reality these rights are often only accessible to those who can afford them.

### Story 3: It is not simply the cost of school fees

While school fees at private schools are high, it is not the only expense. In addition, there are therapy costs, additional childcare costs, medical costs and other considerations. For example, because of developmental delays, toilet training takes much longer (Richardson 2015). Progress through the school system may also have to be extended: one cannot blithely assume that the special needs child will advance along with his or her age cohort despite this being a policy imperative (Department of Education 1996).

Costs could be arranged into three categories: direct medical, direct non-medical and indirect productivity costs (Fletcher et al. 2012:50; Leigh et al. 2016:2).

In addition to school fees, we also relocated to be closer to English-medium schools serving children with special needs. In Tshwane, these schools are concentrated in Pretoria East. That happens to be the upper middle class area of Tshwane, where house prices are considerably higher than elsewhere in the city. The schools are in this area because this is where the people who can afford to pay live. And others, like us, will relocate to the area with appropriate schools, even if they cannot really afford to.

Over the past 7 years, we have worked with six different speech and language pathologists (speech therapists) and three occupational therapists. Our son has received other therapeutic interventions such as Auditory Integrated Training, and various learning integrated therapies. During 2014 in a single month when our medical aid informed us in the middle of the year that our funds were exhausted, we received a therapy account (excluding school fees) of over R13 000. In a study conducted in the USA, Shimabukuro et al. (2008:546) found that the average medical expenditures for children with autism were 4.1–6.2 times that of children without autism.

According to existing research, having a pet dog has a number of benefits for children with autism (e.g. see Hall et al. 2016). In our experience, this has turned out to be true. Our lively Jack Russell Terrier is our son’s constant companion, at the receiving end of verbal instructions, recriminations and blame for any mess or breakage. In short, she has aided our son’s language development, social skills and imaginative play. Our son: ‘Sofia is not naughty! Sofia is cute. Sofia is best friend!’

The dog has also primed our son for therapy (cf. Hall et al. 2016). Yet we would not have had the dog if it was not for our son, and she is another expenditure. The veterinary facilities in this area put human hospitals to shame and are priced accordingly.

While our son is not on medication (at our own insistence), this can be a significant additional cost as noted by Shimabukuro et al. (2008:550), especially if medical aid schemes insist on generics that do not agree with some children. More than once, teachers and school administrators with no medical qualifications have hinted that we should consider medication. But there it has remained. Our son’s problems are not behavioural; in fact, he tends to be a model citizen at school.

**Author 2**: ‘Other parents have been less fortunate and “What is your child on?” is a common topic of conversation outside the school gates, soon followed by “And what are you on?”. One can add the cost of medication to the general cost of raising a child with autism.’

In our case, a significant additional cost has been dental care (cf. Du et al. 2016). Instead of biannual check-ups at a dentist’s surgery, we need to book into a private clinic to have routine check-ups under anaesthetic. This has averaged R10 000 annually with a separate anaesthetist’s bill and a compulsory co-payment.

### Story 4: How deep are your pockets?

During an assessment feedback session, we asked the head of a therapy centre, ‘how many sessions do you recommend for our son?’ We received the response, ‘how deep are your pockets?’, followed by a recommendation for ‘as much as possible, as often as possible’.

**Author 1**: ‘I immediately replied, “Our pockets are as deep as they need to be. We will pay anything we need to. It will simply be our priority”. However, they could not tell us how much would be enough. They were extremely vague on what exactly they will focus on.’

At this meeting, we also signed a legal disclaimer that we would not sue the school if the therapy was not effective. We later heard that the school had recently won a protracted high court challenge by a family who was dissatisfied about their child’s progress. It also reinforced that the therapy centre was primarily a business protecting its own interest.

At the time, because we were aware of the importance of early intervention, we signed up for five sessions of speech therapy per week (one per day) and three sessions of occupational therapy. This amounted to at least 32 sessions per month.

It therefore came as a shock when our son had a 3-day-long pre-admission assessment at his next school 2 years later for which we were charged R2500, and the report read ‘Parents claim that child has received 32 sessions of therapy per month … no evidence of previous therapy’. While this unleashed numerous questions related to how he was assessed, we were deeply troubled that the therapy might have been a waste of time and money. The fact that children are re-assessed on every move to a new school, even though an official diagnosis is demanded, shows that there is little coordination or professional regard between the people involved. It is a clumsy, wasteful system that once again raises the expense of educating a special needs child.

**Author 1**: ‘These schools are completely unaccountable! We did not even receive written feedback on the assessment. On my request, the secretary innocently made a photocopy of the assessment record but it [*was*] not usually given to parents. When I told the principal that I had a copy, she was furious. How can we monitor them if we have nothing in writing? I feel very uncomfortable about this.’

We believe that therapy can be effective. We have come to regard the effects of therapy as cumulative, as results are only evident after several years (Chasson et al. 2007). However, only our sixth (and current) speech therapist is highly skilled, although we only realised this when we compared what she does to what the previous therapists did, and most importantly, our son’s progress. But then, what were we paying for all the years before?

### Story 5: Assessment feedback meeting – Can our son move on to Grade 1 next year?

We often question what makes ‘special schools’ special? Is it their approach, curriculum, specialised teaching methods or specialised teaching skills? What exactly are we paying them for? We are always well prepared for assessment feedback meetings. We analyse each report, compare it to previous reports and agree on a set of questions that we will ask. The main question for us in early 2016 was whether our son would be ready to progress to Grade 1 by the end of that year and take on the formal curriculum, even if only with extra support. The teacher replied,

‘It does not depend on me. I will not be involved. The Grade 1 teachers will decide and there will have to be a formal school readiness test.’

**Author 1**: ‘This simply confirmed what we already suspected – that the teacher is inadequately trained, even though the fees are the highest we’d ever paid. We are being prepared for yet another pricey re-assessment. She often makes remarks concerning his progress, usually related to some new skills that she has recently observed. These were the same things he’s been doing for years at home. School is supposed to teach new things, not only recognise them years later.’

As a parent-practitioner, Author 1 has realised that teachers often underestimate what children can do, and as a result, fail to stimulate and challenge them appropriately.

**Author 2**: ‘There was a more fundamental issue involved. We understand that progression from the special needs programme to the regular, though still additionally supported, grade system would have to involve a team approach to assessment and could not rely on a single individual. But when that individual is the child’s class teacher, who has been with him, six hours a day for over a year, how can she not be involved in the team at all?’

There were internal power relations between school management, the special needs teachers, the regular teachers and the therapeutic team that, in our estimation, took precedence over the best interests of the child. By the following week we had given the school written notice but it would be 3 months before we could move, as prescribed by our contractual obligations. It was after all, first and foremost, a business. But from that day onwards, we gave up on the school, and on the idea of ‘special’ schooling, and enrolled our son in a small private school that caters to both special needs and typically developing children. Here he studies the official curriculum, albeit with much extra support, and is exposed to peers from whom he can really learn.

**Author 1**: ‘I wonder how special schools get away with operating the way that they do. Do parents evaluate schools on the basis of value for money? Are we getting what we are paying for? How will we know? Or do we assume that the greater our financial sacrifice, the better chance we are affording our children?’

Let us look at this from another angle. We have spent approximately 10% of our after-tax income on our son’s education and therapy in 2015. But we have resisted the temptation to subject him to applied behavioural analysis (ABA), an intensive neo-behaviourist form of therapy, not only because of the cost but because we as scholars disagree with the method (cf. Dawson 2003). It is one of the most popular methods available in South African private special schools and is extremely expensive. No ABA institution was prepared to give us an upfront estimate, but their recommended 40 h per week of therapy, at medical aid rates of R440 per hour, works out to R17 600 per week or R704 000 per year for a 40-week academic year.

If we apply our estimate of 10% of our income to the annual cost of ABA therapy, we would require an after-tax income of R 7 040 000 per annum. In 2011, it was reported that just over 2000 South Africans earned more than 5 million rand per year – the top tax bracket, also known as the Y group (Anon 2011; Sapa 2011).

Now consider that a survey by the Bureau of Market Research (2011) at the University of South Africa regards 10.1% of the population as ‘affluent’, a category that in 2011 started at an annual income (before tax) of R1 329 845. The Quest Salary Survey for 2014–2015 showed that the median pre-tax monthly salary for a chartered accountant with less than 5 years of experience (the most likely case for a parent of a young child) is just R42 500, and this was the most lucrative of the many job descriptions covered by the survey (Makhubele & Quinan 2014). In South Africa, even a chartered accountant cannot expect to pay the enormous fees charged for ABA.

The reality is that South Africa has one of the highest Gini coefficients in the world. Around a quarter of the working-age population is unemployed and grinding poverty is all too common. It is important to note that in this particular article, we do not intend to debate on the efficacy or ethics of ABA. What we wish to make clear is that its current implementation in South Africa is not financially feasible for all but the most affluent members of society. We have to consider that most of these are commercial enterprises that will sell you as much therapy as possible without providing any guidance regarding which intervention is needed for a child, which has also been noted by Al Jabery et al. (2014:475).

## Conclusions

South Africa is a signatory to a number of international conventions and its internal policies promote the concept of inclusion. It therefore cannot leave the education of its citizens with autism to the private sector, where only the privileged few will benefit. Nor can it be left to the initiative of an individual family. From the perspective of Critical Autism Studies, we need the joint involvement of the medical and educational establishments backed by the state. Education is about transformation, about optimising the abilities of all learners, including those with autism.

South Africa urgently needs cost-effective early intervention options that can provide meaningful educational opportunities for all in an inclusive setting. Such options can be created, and in this respect India seems to be ahead of South Africa (Brezis et al. 2015; Vaidya 2016). However, directly importing intervention programmes from India seems no more likely to result in a system appropriate to South Africa than importing it from the USA has been. The hard work needs to be done here.

There is also a need for government to regulate the private providers operating in the field. This should include registration, accreditation and mandatory training programmes. There should also be an ombudsman for accountability purposes. As long as parents have no recourse when they are dissatisfied with services provided, they will remain vulnerable to exploitation, especially because private providers are not held accountable for providing quality and meaningful educational services and support for children with autism.

### Limitations of the study

The limitations of this study are linked to its methodology. A single case study, whether autoethnographical or not, cannot give a definitive answer to a research problem. It can, however, point out the direction that future research with different methodological strategies must take.

### Recommendations

We offer the following advice to parents: insist that schools and therapy centres motivate for the number of sessions required. Therapists seldom tell parents that a child has had sufficient therapy. Make sure that therapists can explain their support plan and strategies including exactly what they are working on, how and why. Also ensure that you initiate follow-up review meetings if they do not. Trust your instincts in this regard and assess your child’s progress for yourself, against the goals previously set.

For educators, researchers and policymakers, however, our recommendations are that we urgently need to examine the underlying assumptions of existing intervention strategies and create new, affordable ones that can be rolled out to more than just the urban elite.
